# Extremophilic microbial metabolism and radioactive waste disposal

**DOI:** 10.1007/s00792-023-01312-4

**Published:** 2023-10-15

**Authors:** Sarah Jane Butterworth, Franky Barton, Jonathan Richard Lloyd

**Affiliations:** Department of Earth and Environmental Sciences, Research Centre for Radwaste Disposal and Williamson Research Centre, The University of Manchester, Manchester, UK

**Keywords:** Polyextremophiles, Geological disposal facility, Radioactive waste, Radiation, Bentonite, Cement

## Abstract

Decades of nuclear activities have left a legacy of hazardous radioactive waste, which must be isolated from the biosphere for over 100,000 years. The preferred option for safe waste disposal is a deep subsurface geological disposal facility (GDF). Due to the very long geological timescales required, and the complexity of materials to be disposed of (including a wide range of nutrients and electron donors/acceptors) microbial activity will likely play a pivotal role in the safe operation of these mega-facilities. A GDF environment provides many metabolic challenges to microbes that may inhabit the facility, including high temperature, pressure, radiation, alkalinity, and salinity, depending on the specific disposal concept employed. However, as our understanding of the boundaries of life is continuously challenged and expanded by the discovery of novel extremophiles in Earth’s most inhospitable environments, it is becoming clear that microorganisms must be considered in GDF safety cases to ensure accurate predictions of long-term performance. This review explores extremophilic adaptations and how this knowledge can be applied to challenge our current assumptions on microbial activity in GDF environments. We conclude that regardless of concept, a GDF will consist of multiple extremes and it is of high importance to understand the limits of polyextremophiles under realistic environmental conditions.

## Introduction

Viable microorganisms have been identified in both natural and engineered extreme environments previously considered to be sterile, including highly radioactive areas of the Sellafield nuclear site (Foster et al. 2020) and Chernobyl (Niedrée et al. 2013). The survival of microorganisms in other engineered extreme environments must be considered, including in deep, underground geological disposal facilities (GDF) for higher activity radioactive waste. A GDF offers isolation of radioactive waste from the environment through a multi-barrier system, with each barrier possessing properties that will aid in the reduction of radionuclide migration to the biosphere. Some barriers also function to minimize microbial activity. Concepts differ between countries but can be generically defined by their geology as a high strength rock, low strength sedimentary rock or an evaporite system. Concepts may be further defined by their engineered barrier/backfill material (bentonite, cement, or salt), the choice of which is influenced by the chosen geological environment.

Microorganisms may be introduced into a GDF through anthropogenic contamination during construction, natural geological/hydrogeochemical processes, or be indigenous to the chosen site. Microbial communities may be stimulated by physiochemical changes during GDF evolution (Leupin et al. 2017) and resulting microbial activity could lead to biogeochemical interactions and environmental changes which may have either negative (e.g. acceleration of corrosion of GDF infrastructure e.g. waste containers (Pedersen et al. 2000)) or positive impacts (e.g. reduction of radionuclide mobility (Newsome et al. 2014)) on safety cases. There are still many uncertainties regarding the full potential of GDF microbial communities as their diversity and metabolic capacity are difficult to constrain given the geological timescales involved and the geochemical complexity of a GDF. However, we can begin to constrain metabolic capabilities with GDF environments through our current understanding of microbial adaptations to extremes in a wider range of analogous environments (Butterworth et al. 2021). This review explores the extremes that microbial life may have to overcome in a GDF and what adaptations may allow them to colonise such an environment. A range of relevant GDF studies are discussed, and difficulties in constraining microbial life under in situ conditions are highlighted.

### GDF Evolution—the formation of an extreme environment

All physical, chemical and biological changes greatly depend on the GDF concept specifications and waste inventory, which varies from country to country (NDA 2010). Discussion of site-specific evolutions has been detailed previously (Arcos et al. 2006; NDA 2010; Kosakowski et al. 2014). All concepts will include some form of inherent biological control, whether direct or indirect. Candidate barrier materials considered in this work are detailed in Table 1. Although not an extensive review of all candidate materials, these materials are anticipated to have a significant impact on microorganisms by providing an inhospitable environment. Examples of how different concepts may evolve to reduce microbial activity are given below.

**Table 1:** A summary of the candidate barrier materials discussed in this work with their intended waste group and use

Candidate barrier material	Use	Waste group
Evaporite	Host rock, buffer/backfill (crushed host rock)	HLW/ILW
Bentonite	Buffer/backfill	HLW
Cementitious material	Buffer/backfill	ILW

*HLW*  high-level waste, *ILW* intermediate level waste. Information taken from RWM, (2016)

The post-closure phase refers to the period in which all waste has been emplaced and the GDF sealed. During the first few hundred to tens of thousands of years post-closure, heterogeneous evolution of engineered barriers will begin, mainly caused by heat generation from waste and resaturation of the GDF with groundwater. Resaturation with deep groundwater will lead to the formation of redox gradients and alter geochemical parameters such as pH (Beattie and Williams 2012). Gas generation from corrosion, radiolysis and microbial degradation or organics in the wastes will also impact on physiochemical evolution (Towler et al. 2012). Not all barrier evolutions are negative as some are proposed to enhance GDF safety requirements, such as reduction in hydraulic permeability through swelling of materials (Sellin and Leupin 2014).

Bentonite clay contains the phyllosilicate mineral montmorillonite, which swells upon contact with water. The swelling of montmorillonite is the main safety feature of a bentonite barrier, generating a high pore pressure that provides lower permeability and porosity (Hicks et al. 2009). Understanding bentonite barrier evolution is important for controlling microbial activity, which is anticipated to be inhibited by swelling pressures. Heterogeneity or changes over time in the bentonite barrier may allow for microbial communities to develop. Of particular interest are sulphate-reducing bacteria (SRB), which produce the corrosive species hydrogen sulphide that may compromise metal waste container integrity (Pedersen 2010).

In a cementitious GDF, geochemical evolution is driven by cement dissolution stages, which have been previously detailed by Berner (1992). Extensive use of cement in a GDF will result in hyper alkaline conditions (pH 12–13) after the intrusion of groundwater, limiting microbial activity and is a key safety feature of a cementitious GDF concept. Over time, the overall pH of the GDF will decrease as cement dissolution progresses, reducing from an initial maximum pH of 13.5 to the pH of the incoming groundwater in the final stage of the evolution (NDA 2010). At what point in the GDF evolution alkalinity becomes low enough to support microbial life on an impactful scale is of interest to the safety case and requires further constraints on life at high pH. This is important as microbial activity may lead to the degradation of highly soluble cellulose-derived products that may be complex with radionuclides (such as isosaccharinic acid (ISA)) and also contribute to gas generation and degradation (Small et al. 2017).

Salt rock, one potential GDF host geological formation, has the ability to ‘self-heal’ after a few decades of closure (Tsang et al. 2005). Self-healing capabilities are a result of the elastoplastic behaviour of salt, which is reported to accelerate void closures (Brewitz and Rothfuchs 2007). Self-sealing will prevent the intrusion of groundwater (Hunsche and Hampel 1999; Brewitz and Rothfuchs 2007) and consequently result in a dry environment with very low rates of fluid flow, limiting microbial activity. Brine pockets and intergranular water accumulated prior to self-sealing are predicted to be the main fluid sources, which may migrate towards the canister as a result of thermal activity (Tsang et al. 2005). Microbial life in a post-closure salt environment is less studied in comparison to other concepts, but scientific interest remains around microbial-radionuclide interactions in this extreme environment (Bader et al. 2017).

Microorganisms may have both positive and negative impacts on GDF safety cases across all concepts. Operational activities may provide colonisable space in the form of fractures and anoxic groundwater saturation will lead to the formation of a redox gradient, which may allow for various types of microbial respiration to take place. The complexity and scale (spatial and temporal) of a GDF will inevitably lead to heterogeneities that will allow for small-scale microbial activity. Nevertheless, the post-closure GDF is an extreme environment that provides many physical and chemical challenges to microbial life.

### GDF extremes

Examples of anticipated GDF extremes based on modelling, laboratory and in situ tests are presented in Table 2, though it should be noted that these may not represent the upper limits found in a GDF. For example, the initial pH of cementitious pore water could theoretically be higher than pH 12.7 (Berner 1992).

**Table 2:** Examples of extreme geochemical parameters anticipated in different GDF concepts, based on laboratory, in situ and modelling techniques

Parameter	Anticipated measurement	GDF Concept	References	Upper Limits	References
Pressure (MPa)	5*	Swedish HLW model (bentonite)	Bengtsson et al. 2015	125	Dalmasso et al., 2016
pH	12.7	Swiss LILW model (cement)	Wieland et al. 2020	12.5	Suzuki et al., 2014
Salinity (%)	9^#^	Canadian HLW model (bentonite)	Stroes-Gascoyne et al. 2008	35	Oremland et al., 2005
Radiation (Gy/h)	0.5^+^	Swedish HLW model (bentonite)	SKB 2006	30	Brown et al., 2015
Temperature (°C)	100, 125 and 200^&^	Host rock: high integrity > sedimentary > evaporite	NDA 2016	122	Takai et al. 2008

Note that these are subject to change as these do not represent the final GDF conditions. *HLW* high level waste, *LILW* low intermediate level waste. * Target bentonite swelling pressure at full water saturation, achieved via a clay wet density of approx. 2000kgm-3. # Salinity of groundwater from the Atomic Energy of Canada Limited’s Underground Research Laboratory at a depth of 420m. + Calculation of the maximum dose outside the canister, taking into account buffer attenuation. & Temperature limits are defined based on host rock integrity

The reported limits for microbial life vary in the literature. The definitive values may depend on cultivation techniques and what is considered by the author to be the upper limit of life (e.g., optimum growth or tolerance limit). The limits of life in pure cultures are better studied but may be different to those achieved within the potentially less favourable GDF environment.

When compared to the known upper limits of life in pure culture experiments (referring to Merino et al. 2019), individual geochemical parameters within a GDF are less severe. However, a GDF is an environment that will have multiple stresses acting upon the microbial community at the same time, adding further constraints for microbial life. The limit of life in a GDF is therefore difficult to determine based on one extreme, regardless of whether it is defined by optimum growth or tolerance. Understanding extremophilic adaptations to individual extremes is still useful, however, as it may reveal complimentary adaptations that would aid survival in a poly extreme environment.

### Microniches

If microbial life is to become established in a GDF, it is likely to do so in a “microniche” where conditions are more favourable. The formation of microniches is dependent on the heterogeneity of engineered barriers, especially those resulting from GDF evolution. In this context, a microniche is defined as an area with increased biogeochemical activity in comparison to the large-scale environment and is confined to pore spaces, cavities, fractures, and boundary zones. In a cementitious GDF, hyperalkaline conditions (pH 12 – 13) will limit microbial activity but pH is not anticipated to be consistent throughout the GDF and areas of lower pH will exist (Askarieh et al. 2000). For example, high pH (pH 11) microbial activity (see Alkalinity section) has also been observed to lead to a decrease of pH in the Finnish large-scale Gas Generation Experiment (Small et al. 2017), through the production of volatile fatty acids. These areas of lower pH, in an “alkaline disturbed zone”, provide a more favourable environment and may allow for the colonisation of microbial communities within a GDF in the form of microniches (Rizoulis et al. 2012). Bruhn et al. (2009) have also suggested that anaerobic bacteria would be capable of forming anoxic microniches within a repository environment. The lack of oxygen in an anoxic microniche could contribute to increased radiation tolerance in anaerobic bacteria due to the lack of oxygen atoms available to react with ionising radiation and form reactive species that damage cells.

Furthermore, microbial consortia may exist interdependently in a biofilm, a community structure considered to be representative of a type of microniche (Costerton et al. 1994). Biofilms consist of extracellular polymeric substances (EPS), such as polysaccharides, proteins, and lipids, which assist in survival and can provide protection in harsher environments (Yin et al. 2019). EPS serves many functions, including nutrient acquisition via sorption and retention (Sutherland 2001). Nutrient retention is a particularly important feature in low-nutrient environments such as GDFs, where biofilms are likely to exist where nutrients are locally available. In radioactive waste disposal, research has focused on the impact of biofilm growth on canister materials including steel and copper (Huttunen-Saarivirta et al. 2016, 2018). In biocells under high pH conditions, biofilms have been observed to form in cement and on graphite and steel surfaces, provided an initial floc community for formation (Charles et al. 2017a, 2017b). There is also growing interest regarding how microorganisms may alter minerals within bentonite. Smectite-biofilm interactions have shown biofilm contribution to mineral dissolution and redox changes (Perdrial et al. 2009).

High-resolution techniques are required to identify microniches in the field (Stockdale et al. 2009) and in the closed, deep environment of a GDF, monitoring of the development of these microniches will be limited. URLs and associated modelling can be used to better understand microniche development as there are less access restrictions. Microniche distribution is also difficult to predict due to the many potential interactions that may underpin their development, and high-resolution techniques are required to monitor activities across very small (micron) scales within highly complex matrices. The importance of understanding the pH limits of microbial activity, heterogeneity, and the formation of microniches within a high pH GDF is critical to the safety case and has been demonstrated in analogue biofilm systems (Charles et al. 2022).

### Nutrients and energy sources

A main consideration for life in the deep subsurface GDF is the quantity of organic carbon available to be utilised as a carbon source and an electron donor. Natural concentrations of organic carbon in various GDF environments are summarised in the work of Reiller et al. (2002). Unsurprisingly, the groundwater in sedimentary environments is richer in natural organic matter than in granitic environments (Reiller et al. 2002). During the construction of a GDF, organics (e.g., synthetic fibres, hydrocarbons from rock drilling) are expected to be introduced anthropogenically in low quantities (Marshall and Simpson 2014). Specific GDF concepts may further contribute to the organic carbon content. For example, ILW may contain substantial quantities of cellulosic materials and the natural organic matter of clay will also contribute to overall organic concentrations in bentonite concepts (Marshall and Simpson 2014).

Despite the introduction of further sources of organic matter to the GDF, the concentration of dissolved organic carbon is still anticipated to be low. Interestingly, microbial community dependency on organic carbon can depend on residence time and mixing of groundwater types. In a borehole study within the Äspö Hard Rock Laboratory, Sweden, waters with a marine origin displayed a greater dependency on organic carbon in comparison to older saline waters, where a greater dependency on geogases such as hydrogen was observed for microbial metabolism (Lopez-Fernandez et al. 2018a, b). Lopez-Fernandez et al. (2018a, b) observed the rapid recycling of organic carbon from the degradation of non-viable cells, providing evidence as to why dissolved organic carbon is so low in the subsurface (Itävaara et al. 2016). Limitations for understanding energy and nutrient availabilities lie with the difference between laboratory experiments and conditions in situ in the deep subsurface. Using SRB as an example, microbial communities in laboratory experiments are observed to have much shorter carbon turnovers (< 1 year) in comparison to in situ communities (> 10 years) (Hoehler and Jørgensen 2013).

The intrusion of anoxic groundwater into a post-closure GDF will lead to the formation of a redox gradient. Early in a GDF’s lifecycle, oxygen will remain from construction and as the most favourable terminal electron acceptor, aerobic respiration will occur until it is depleted. As conditions become more reducing, the following electron acceptors are anticipated to be utilised in the following order, if present: nitrate (in the waste form itself (Bleyen et al. 2018)) iron (e.g. from bentonites (Kim et al. 2004), corrosion of engineering infrastructure and waste containers) and sulphate (from sulphate-bearing groundwaters and sulphate present in bentonites).

With organic matter limited in some concepts, microbial life will likely be restricted to chemolithotrophs with a reliance on hydrogen as an electron donor. Libert et al. (2011) have previously summarised evidence that hydrogen (produced by processes including corrosion and radiolysis) can support microbial life in a GDF. Further work by Lopez-Fernandez et al. (2018a, b) details hydrogen-driven carbon cycling in the deep subsurface aquifers of the Äspö Hard Rock Laboratory. However, the extent of microbial activity will likely be limited by other factors, such as phosphorous and nitrogen availability.

Lack and/or depletion of nutrients will likely result in starvation and resource limitation for microbial communities. To ensure survival, some bacteria may enter a state of dormancy through a reallocation of resources, known as a stringent response. Several methods of dormancy exist and are summarised by Lennon and Jones (2011). Although dormancy in a GDF environment is not limited to spore-formers (dormant prokaryotes have been successfully cultured from ancient halite (Gruber et al. 2004)), spores are focused on in this work due to their durability and relevance across various GDF concepts.

Even if microorganisms are inhibited during the harshest phase of GDF evolution, viable spores are likely to be present due to their persistence and easy dispersion. An example of these characteristics has been demonstrated by studies of thermophilic spores discovered in the permanently cold marine sediments of Aarhus Bay, far from the warm environments from which they must have originated. These spores have an estimated spore abundance half-life between 250 and 440 years (De Rezende et al. 2013).

Key spore formers of interest in GDF studies include novel isolates from natural analogues, such as the isosaccharinic acid degrading alkaliphile, *Anaerobacillus isosaccharinicus* (Bassil and Lloyd 2019) and a range of SRB and iron-reducing bacteria (IRB) (Williamson et al. 2013). Spore presence is discussed in most papers regarding microbial clay communities, as several bentonites selected for engineered barriers contain indigenous spore-formers (Haynes et al. 2018; Matschiavelli et al. 2019; Grigoryan et al. 2020; Podlech et al. 2021). Clay materials have the capacity to act as ‘microbial seed banks’ (Lennon and Jones 2011), containing dormant individuals that may be resuscitated when the environment permits (Lennon and Jones 2011). Several putative spore-forming IRB have been identified in even commercially available bentonite (Gilmour et al. 2021) and have been found to survive the high temperature and water-limited conditions expected at the host rock/clay interface (Gilmour et al. 2022). Iron reduction by IRB and sulphate-reducing prokaryotes (SRP) is implicated in altering the physiochemical properties within bentonite (Pentráková et al. 2013; Miettinen et al. 2022) and increasing corrosion rates of waste steel canisters (Schu et al. 2015). SRB spore populations of commercial bentonites have been observed to remain viable despite exposure to stressors including high temperatures which active bacterial cells would not be able to survive (Haynes et al. 2018). SRB are also implicated in waste canister corrosion through the production of hydrogen sulphide (Hajj et al. 2010; Hall et al. 2021). There is however debate regarding the importance of spore-formers under continuously low energy conditions, mainly due to the energetic cost of germination to a vegetative cell and the associated repair of macromolecules (Hoehler and Jørgensen 2013). Miettinen et al. (2022) found that the inherent dissolved organic carbon within bentonite was able to sustain microbial activity of dominantly IRB and SRP spore formers for less than two years, after which electron donor additions were required to sustain growth within anoxic microcosms.

The following sections address how our understanding of extremophiles from natural microbial communities can be applied to understanding the limits of life in a GDF, addressing each key geochemical parameter individually. Understanding gained from GDF-specific studies and current limitations are also discussed.

### Radiation

High levels of ionising radiation will result from the beta decay of fission products (e.g. ^90^Sr), alpha decay of actinides (e.g. ^235^U) and gamma rays in HLW concepts (Ewing et al. 1995). In some scenarios, barrier materials are estimated to receive doses of up to 72 Gy/h, although doses will decrease with time (Allard & Calas, 2009). Such doses of ionising radiation will be a controlling factor in long-term microbial metabolism, due to the production of reactive oxygen species (ROS) from the radiolysis of water (Daly 2012). ROS causes cellular structural damage through the oxidation of DNA, RNA, lipids and proteins, a process known as the indirect effect (Desouky et al. 2015). To survive the indirect effect, microorganisms will need to successfully scavenge and neutralise ROS. The evolution of proteins (such as superoxide dismutase) and the production of low molecular weight compounds (metabolites including glutathione, ascorbate and trehalose) can serve to sequester ROS by acting as endogenous scavengers (Salin and Brown-Peterson 1993; Jin et al. 2019).

The indirect effect has been observed to damage the DNA of both radiation-resistant and radiation-sensitive microorganisms. However, radiation-resistant microorganisms are able to survive hundreds of DNA double-bond breakages induced by ionising radiation (Daly et al. 1994), suggesting that ionizing radiation-induced death is not mainly mediated by DNA damage (Daly et al. 2007; Fagliarone et al. 2017; Park et al. 2017). Daly (2009) proposed that ionising radiation-resistant organisms that can minimise protein damage are able to retain enough DNA-repair function, thus maintaining the ability to restore chromosomal damage which would otherwise lead to cell death. Examples include *Deinococcus radiodurans,* a radiation-resistant bacterium that has been the focus of many radiation studies. *D. radiodurans* was first isolated from radiation-sterilised meat containers by Anderson et al. (1956) and closely related isolates were discovered in the high-level radioactive waste plume at Hanford (Fredrickson et al. 2004). It is thought that *D. radiodurans* can survive under such ionising conditions due to the production of Mn antioxidants, with observations in several species of bacteria showing that a higher Mn:Fe ratio improves resistance to IR-induced protein oxidation (Daly et al. 2004). Other protective antioxidants produced by *D. radiodurans* include carotenoid deinoxanthin, which has been found to express anticancer activity as well as very effective ROS scavenging capability (Choi et al. 2019). Shuryak et al. (2017) observed that when cultured with *D. radiodurans*, *E. coli* is more likely to survive when exposed to chronic ionising radiation (CIR). The secretion of antioxidants into the medium by *D. radiodurans* is considered to provide a collective radioprotection. It is possible that a similar collective radioprotection could occur in GDF scenarios, although to date no investigation is known to have been published. The biosorbent properties of *D. radiodurans* are also being considered for the treatment of heavy metal and radionuclide pollution (Guo et al. 2022), emphasising the potential role of radiation-resistant microorganisms in limiting radionuclide mobility in a GDF scenario.

It is hypothesised that radiation resistance is a consequence of adaptations for desiccation. Mattimore and Battista (1996) suggest there is no need for direct radiation adaptation in the natural environment, as levels of ionising radiation are only introduced anthropogenically. The further work of Rainey et al. (2005) supports this argument through the observation that several bacterial species (including members of the *Deinococcus* genus) originating from arid soils exhibit ionizing radiation-resistance. As the Hanford site is in a desert environment, its arid conditions could have selected for *Deinococcus,* rather than the radioactive plume.

If a GDF microbial community contains desiccation-resistant microorganisms, there is a chance for continued microbial activity. Desiccation-resistant bacteria have been identified within clays considered for a GDF (Pedersen 2002; Horn et al. 2004; Mulligan et al. 2009; Vachon et al. 2021). Experiments investigating a stimulated clay-host rock and clay-canister barrier found that desiccation-resistant *Deinococcus radiophilis* survived the furthest (3–6 mm) into the clay (SKB 1999). In an early post-closure GDF, ionising radiation exposure will be continuous over an extended time (chronic radiation). Even so, radiation studies generally rely on intense radiation with short exposure times (acute radiation), as long-term lower-dose radiation laboratory experiments representative of GDF conditions are very challenging. How applicable acute radiation studies are to chronic radiation exposure is unclear. Chronic radiation studies are rare, but a study by Shuryak et al. (2017) found a weak relationship between acute and chronic radiation resistance, suggesting that extrapolating results from acute to chronic radiation scenarios may not be reliable. Radiation may also indirectly influence microbial activity by increasing the bioavailability of Fe(III) through structural changes (disaggregation and increased crystallinity) and increasing organic acids through radiolytic degradation of cellulosic material (Brown et al., 2014, 2015). Finally, it is worth noting that Enyedi et al*.* (2019) found unexpectedly high microbial diversity within biofilms, which appeared to be adapted to the high radioactivity within natural hydrothermal spring caves in Hungary.

### Salinity

High salinity groundwaters may be found at repository depths. In the UK for example, deep groundwater salinity sources include intrusion of seawater, dissolution of evaporites such as halite (Younger et al. 2015) and upconing of deep saline waters with complex origins (Bath et al. 1996). All GDFs have the potential for varied levels of salinity, not just salt-based concepts.

Hypersaline environments cause microorganisms to lose water and cellular structure due to high osmotic pressure and frequent desiccation. Halophiles have adapted to survive high osmotic pressure in various ways, depending on the required salinity tolerance. For example, a halophile may accumulate large concentrations of salts within their cytoplasm to reach equilibrium with the salt concentration of their environment (Edbeib et al. 2016). Accumulation of compatible solutes (low molecular weight molecules, such as sucrose) can aid in the balance of osmotic stress and maintenance of protein structure and stability without being problematic for essential cell processes (Poolman and Glaasker 1998). As high salinity affects the solubility and structure of proteins, halophiles also have modified enzymes to adapt to their environment (Zhang et al. 2013). Oxygen availability is a further limitation of saline environments, due to low solubility in salt-saturated brines. Archaeal halophiles have adapted by producing gas vesicles or aerotaxis sensors that allow them to detect the water–air interface, whilst others can survive anaerobically (Hou et al. 2000; Andrei et al. 2012).

High salt content of hypersaline environments results in periodic evaporation, which exposes halophilic cells to desiccation and DNA damage through double-stranded breaks (Potts 1994). Kottemann et al. (2005) observed that the archaea *Halobacterium* sp*.* could also withstand DNA damage from gamma irradiation (D_10_ = 5 kGy), thought possible due to the adaptation to survive in a hypersaline, desiccated environment. High concentrations of internal salts provide the availability of an endogenous hydroxyl radical scavenger, mitigating the effects of ROS (Salin and Brown-Peterson 1993). In addition, *Halobacterium* sp*.* possesses similar non-enzymatic Mn antioxidant systems to *D. radiodurans* (Webb et al. 2013). In saline solutions, it has been shown that some •OH will be scavenged by chloride, forming perchlorate ions (Büppelmann et al. 1988; Kelm and Bohnert 2017) which may fuel the metabolism of perchlorate-reducing bacteria (Coates et al. 1999; Youngblut et al. 2016). Abiotic scavenging of ROS by chloride, in the same way as intracellular scavenging in halophiles, may decrease the oxidative stress imposed in saline groundwater.

In GDF studies, most microbial work regarding salinity is focused on the impact of microorganisms within a salt rock concept, where salinity is the most obvious stress to overcome. The Waste Isolation Pilot Plant (WIPP), a salt-based GDF in New Mexico, USA, has been central to understanding halophilic communities and their interactions with radionuclides (NEA 2018). Halophiles that have been isolated from WIPP samples include *Halobacterium noricense* and *Chromohalobacte*r sp., which have been observed to adsorb U (Bader et al. 2017) and Np (Ams et al. 2013), respectively. *Halobacterium salinarium* has been observed to adsorb Eu(III) and Cu(III), with results implying a more complex coordination environment than non-halophilic bacteria (Ozaki et al. 2004). Overall, the concern regarding the impact of microorganisms in salt rock is low due to hypersalinity limiting the structure and functionality of microbial communities (NEA 2018).

Salinity is dependent on the GDF concept and associated groundwaters. Although salinity is much less extreme in cementitious and clay GDF concepts, the impact of salinity should not be overlooked. In natural high-pressure environments such as the deep sea, it is hypothesised that it is not pressure that is the limiting factor on microbial life, but the other associated extremes, such as high salinity, radiation (Merino et al. 2019), and temperature (Jørgensen and Boetius 2007), which may also be the case in a GDF environment.

### Alkalinity

Alkaline conditions (which are characteristic of cementitious GDFs) can cause the ionisation of acidic enzymes within a cell, which is one of the reasons microorganisms must maintain a near-neutral cytoplasmic pH (Krulwich et al. 1997). Alkaliphiles have been observed to grow in alkaline conditions above pH 10 (Bassil et al. 2015a, b) and have several adaptive strategies to maintain pH homeostasis. Examples of adaptations include increased metabolic acid production; increased synthesis of ATP; changes to surface properties (e.g. negatively charged cell wall); and increased expression of monovalent proton/cation antiporters (e.g. Na^+^/H^+^) (Padan et al. 2005). Increased expression of antiporters requires rapid recycling of Na^+^ (Krulwich et al. 1997), so alkaliphilic organisms may adapt to accumulate osmoprotective compounds similarly to halophiles (Zavarzin et al. 1999). Extremes of pH are particularly problematic for bioenergetics, and alkaliphiles utilise a sodium motive force rather than a proton motive force to avoid cytoplasmic proton loss (Padan et al. 2005).

For GDF studies, there has been a focus of research on high pH environments resulting from anthropogenic activity, including work done on sediments from Harpur Hill (Derbyshire, UK), a legacy lime workings site. Harpur Hill has a high calcium content and a source of cellulose from the surrounding vegetation (Milodowski et al. 2013), which can undergo alkaline hydrolysis, as anticipated in a cementitious GDF.

High pH microbial work to date has varied from the lab-scale using natural analogue sediments, to field experiments with associated modelling. Within a GDF, the ILW can have many varied types and compositions, including organic material which is largely cellulosic e.g. paper, filters, tissue and cotton. The cementitious GDF conditions and resulting elevated pH and calcium ion concentrations lead to abiotic alkaline hydrolysis of the cellulose to isosaccharinic acid (ISA) (Glaus et al. 1999). Bacteria that can degrade ISA have been identified from high pH natural analogue sediments (Bassil et al. 2015a, b; Kuippers et al. 2015; Rout et al. 2015; Bassil and Lloyd 2019). ISA is highly soluble and may complex with a range of radionuclides (e.g. U, Np) (Gaona et al. 2008), therefore the presence of these ISA degrading bacteria in a GDF scenario has the potential to reduce radionuclide mobility and release to the biosphere.

### Pressure

The interest in pressure tolerance in GDF studies is mainly linked to the impact of swelling pressure on microbial life in bentonite systems, although pressure may also arise from the depth of the GDF and the rock overburden.

The well-studied deep-sea environment has provided much insight into adaptations and coping mechanisms of microorganisms living under high pressures. Jørgensen and Boetius (2007) concluded that pressures of up to 100 MPa were not a deterrent for microbial life. Abe, Kato and Horikoshi’s (1999) work on pressure-regulation in deep sea piezophiles highlighted that as well as the lipid composition of the membrane, respiratory proteins and a membrane-localised signalling system also aids in pressure adaptations. Pressure affects microorganisms both structurally and metabolically in a successive manner, with most microorganisms losing cell viability at 200 MPa. Motility and substrate uptake are among the first processes to be inhibited and protein and DNA structure are the last (Abe 2007). Piezophilic genera produce an abundance of unsaturated fatty acids within their cell membrane to maintain membrane fluidity, which would otherwise be compromised by the application of pressure (Kato 2012). In response to pressure increases, microorganisms are also known to produce organic compounds known as piezolytes, that act to stabilise proteins under high hydrostatic pressure (Papini et al. 2017). Another observed pressure adaptation includes the elimination of cavities in the protein core via decoupling of protein-water dynamics via rearranging protein surface charge residues to use bulkier hydrophobic residues in the core (Caliò et al. 2022). It was previously believed that piezophiles only existed in pressurized environments, however, recent findings support a new hypothesis, that piezophiles have adopted a common adaptation strategy for dealing with multiple types of stressors (Wang et al. 2021).

In clay barriers, the safety function is considered to be met if the swelling pressure is between 0.1 and 1 MPa (Sellin and Leupin 2014), suggesting that microorganisms within the clay will require a level of piezophily (not necessarily including those that colonise interfacial space). Swelling of clay should limit microorganisms by reducing the average pore throat diameter to < 0.05 µM and consequently reducing colonisable space (Leupin et al. 2017). Swelling capacity of clays greatly depends on groundwater composition and is unlikely to be homogenous, so it must be assumed that there may be heterogeneities where microbes can colonise.

In bentonite clay system experiments, the focus is on the impact of swelling pressure on SRB viability (Bengtsson et al. 2015), which if active can cause waste canister corrosion through the production of hydrogen sulphide. Laboratory pressure cell research suggests that a wet clay density of at least 1800 kg m^−3^ is needed to achieve the swelling pressure required to greatly reduce or inhibit SRB activity (Bengtsson and Pedersen 2016; Haynes et al. 2019).

### Temperature

The high activity of wastes arising from spent nuclear fuel may lead to a significant rise in temperature around the canister as a result of their radioactivity. To maintain the integrity of engineered barriers and limit the corrosion of waste canisters, GDF concepts have a temperature limit. Maximum temperature limits are based on whether the host rock is high integrity (100 °C), sedimentary (125 °C) or evaporite (200 °C) (NDA 2016). Based on these limits, microorganisms that could survive close to the canister surface would be classified as hyperthermophiles, organisms that grow optimally in environments 80 °C and above. As there will be a temperature gradient in a GDF, thermophiles and mesophiles may also be expected to colonise the barrier system further away from the canister.

In the natural environment, hyperthermophiles are found in a variety of environments resulting from geothermal and volcanic activity, such as deep-sea hydrothermal vents and hot springs. Hyperthermophiles with optimum growth temperatures of 80 °C and above have been identified in the Guaymas basin (Gulf of California, USA) (Jørgensen et al. 1990) and Yellowstone National Park (Wyoming, USA) (Kashefi et al. 2002). In pure culture, the current limit of life is reported to be 122 °C (Takai et al. 2008; Merino et al. 2019).

High temperatures close to 100 °C denature proteins and increase the fluidity of the membrane to a point that results in cell death (thermal lysis). Thermophiles may adjust fluidity by changing the lipid content of the membrane (e.g. increasing the amount of branched-chain iso-fatty acids) (Siliakus et al. 2017). Hyperthermophiles are able to stabilise their proteins through higher packing densities and decreased volumes to counteract the expansion caused by high temperatures (Sterner and Liebl 2001).

The Full Scale Engineered Barrier Experiment (FEBEX) completed in the Grimsel laboratory (Switzerland) was set up to test the use of bentonite as an engineered barrier based on the Spanish GDF concept, with a focus on the influence of heat on hydrogeochemical processes (Torres et al. 2017). A central heating element was used to generate a thermal gradient expected from an HLW canister and was continuously monitored for 18 years. At the end of the experiment, bentonite cores were extracted and used for microbial analyses, detailed in the report by Bengtsson et al. (2017). Bacteria cultured from FEBEX samples were able to grow at 70 °C, but these culturing techniques only indicate that bacteria had remained viable, not whether they were actively growing during the experiment. The increasing relative abundance of *Cyanobacteria* towards the heater suggests increased inactivity with higher temperatures, as *Cyanobacteria* are photosynthetic and could not have grown in situ (Haynes et al. 2021). Further, viable cell counts (calculated from the most probable number technique) were low near the heating element (Bengtsson et al. 2017).

### Polyextremophiles

From reviewing a range of targeted extremophilic adaptations, it can be considered that some extremophiles are equipped to survive a wider range of extremes that impose similar stresses. For example, as many extremes lead to environmental water stress, it is likely that adaptation to low water activity will be beneficial for surviving multiple stresses. Examples of overlapping adaptations are provided in Table 3.

**Table 3:** Examples of overlapping adaptations for pairs of extremes

Extremes	Example of overlapping adaptation	References
Radiation and salinity	Endogenous ROS scavengers as a result of salt-in adaptation	(Kottemann et al. 2005)
Pressure and salinity	Osmolytes which may act as piezolytes (e.g. hydroxybutryate)	(Jebbar et al. 2015)
Alkalinity and salinity	Rapid recycling of Na^+^ requires the production of osmoprotectants	(Krulwich et al. 1997)
Pressure and high temperature	Pressure stabilising thermostable proteins above 100 °C ^+^	(Robb and Clark 1999)
Radiation and Pressure	Entering a hibernation state protects from pressure extremes and ionizing radiation	(Weronika and Łukasz, 2017)

It is well documented that extremophile may grow optimally under two extreme conditions, but a GDF environment may impose more than two extremes. When considering polyextremophiles that can withstand three or more extremes, few reports exist in the literature. Their absence may be due to the optimisation or tolerance of multiple extremes requiring high energetic costs [for example, resources required to deal with protein and RNA repair (Kempes et al. 2017)]. On the other hand, many extremophiles remain recalcitrant to cultivation (Jørgensen and Boetius 2007; Hammes et al. 2011; Horikoshi 2011), and polyextremophiles may have been overlooked due to lack of cultivation, particularly if they were not the focus of a study.

In this context, the discovery of the halophilic, alkalithermophilic *Natranaerobius thermophilus* from the lakes of Wadi an Natrun, Egypt (Mesbah et al. 2007) has raised interesting questions about the limits of microbial life in the presence of multiple extremes. If a microorganism is utilising adaptive strategies for one extreme (for example, membrane-based), then there are less resources to deal with additional stressors. Bowers et al. (2009) conducted a review of polyextremophiles (including those found in Wadi an Natrun) and found that the more extreme the optimum growth is for one condition (e.g. high temperature), the less extreme the optimum for other conditions (e.g. high pH, salinity) tends to be.

Another polyextremophile, *Clostridium paradoxum* (haloalkaliphilic, moderate thermophile) can grow with a combination of hydrostatic pressure, high temperature and alkalinity, with the highest growth rates measured at 22 MPa, 60 °C and pH 10 respectively. This combination of extremes for optimal growth is unusual, as high temperature and hydrostatic pressure exert opposite effects when applied independently (Scoma et al. 2019) and therefore require different adaptations. Prior to this study by Scoma et al. (2019), there was no known record of *Clostridium paradoxum* being grown under hydrostatic pressure, so piezophily was unknown. This discovery, although currently unique, challenges our assumptions of polyextremophiles and highlights the importance of laboratory and in situ tests to further our understanding of the physicochemical limits of life. Until the response of microbial life to polyextremes in GDF-relevant environments has been assessed, we cannot assume GDF conditions will be harsh enough to constrain metabolic activity.

### Concluding remarks

Microbial activity has been identified as an important consideration in GDF safety cases across various concepts, all of which share common uncertainties regarding the limits, impacts and diversity of microbial metabolism under GDF conditions. The ability of a GDF to support microbial life is largely unconstrained and will be determined by energy and nutrient availability, alongside extremophilic adaptations. By not considering the impacts of multiple extremes, the impact of microbial metabolism may likely be misrepresented in a range of GDF scenarios. A major challenge in understanding extremophiles in GDFs is how to reproduce a representative system with a reasonable scale of complexity. A more realistic environment can be provided via an underground research laboratory (URL), which may be generic or site-specific. Several countries have active URLs, which have proved useful in developing safety cases (NEA 2013). In some cases (particularly with natural analogues), opportunities to better understand microbiology have been lost or are incomplete due to oversight in the initial planning stages, likely in part linked to technical limitations and the challenging nature of the systems. New advances in omic (e.g. genomics) technologies and novel ways to identify key microbial community members (e.g. stable isotope probing) are continuously being refined and are helping drive understanding. It is hoped that dedicated URL microbiological studies continue applying these techniques, with a focus on polyextremophiles, their role in controlling the biogeochemical evolution of GDF scenarios, and the long-term impact of microbial processes on the safe geological disposal of nuclear waste.

## Abbreviations

FEBEX: Full-scale engineered barrier experiment
GDF: Geological disposal facility
HLW: High-level waste
ILW: Intermediate-level waste
IRB: Iron-reducing bacteria
ISA: Isosaccharinic acid
LILW: Low intermediate-level waste
NDA: Nuclear Decommissioning Authority
ROS: Reactive oxygen species
SRB: Sulphate-reducing bacteria
URL: Underground research laboratory

## References

[CR1] Abe F (2007). Exploration of the effects of high hydrostatic pressure on microbial growth, physiology and survival: perspectives from piezophysiology. Biosci Biotechnol Biochem.

[CR2] Anderson AW, Nordan HC, Cain RF, Parrish G, Duggan D (1956). Studies on the radio-resistant Micrococcus. I. The isolation, morphology, cultural characteristics and resistance to gamma radiation. Food Technol.

[CR3] Andrei AŞ, Banciu HL, Oren A (2012). Living with salt: Metabolic and phylogenetic diversity of archaea inhabiting saline ecosystems. FEMS Microbiol Lett.

[CR4] Arcos D, Grandia F, Domènech C (2006). Geochemical evolution of the near field of a KBS-3 repository.

[CR5] Askarieh MM, Chambers AV, Daniel FBD, Fitzgerald PL, Holtom GJ, Pilkington NJ, Rees JH (2000). The chemical and microbial degradation of cellulose in the near field of a repository for radioactive wastes. Waste Manage Pergamon.

[CR6] Bader M, Müller K, Foerstendorf H, Drobot B, Schmidt M, Musat N, Swanson JS, Reed DT, Stumpf T, Cherkouk A (2017). Multistage bioassociation of uranium onto an extremely halophilic archaeon revealed by a unique combination of spectroscopic and microscopic techniques. J Hazard Mater.

[CR7] Bassil NM, Lloyd JR (2019). Anaerobacillus isosaccharinicus sp. nov., an alkaliphilic bacterium which degrades isosaccharinic acid. Int J Syst Evol Microbiol.

[CR8] Bassil NM, Bewsher AD, Thompson OR, Lloyd JR (2015). Microbial degradation of cellulosic material under intermediate-level waste simulated conditions. Mineral Mag.

[CR9] Bassil NM, Bryan N, Lloyd JR (2015). Microbial degradation of isosaccharinic acid at high pH. ISME J Nature Publ Group.

[CR10] Bath AH, Mccartney RA, Richards HG, Metcalfe R, Crawford MB (1996). The geology and hydrogeology of the Sellafield Area Groundwater chemistry in the Sellafield area: a preliminary interpretation. Quarterly J Eng Geol.

[CR11] Beattie TM, Williams SJ (2012). An overview of near-field evolution research in support of the UK geological disposal programme. Mineral Magaz Mineral Soc.

[CR12] Bengtsson A, Pedersen K (2016). Microbial sulphate-reducing activity over load pressure and density in water saturated Boom Clay. Appl Clay Sci.

[CR13] Bengtsson A, Blom A, Taborowski T, Schippers A, Edlund J, Kalinowski B, Pedersen K (2017). FEBEX-DP: Microbiological report, NAGRA NAB 16–15.

[CR14] Bengtsson A, Edlund J, Hallbeck B, Heed C, Pedersen K. (2015) Microbial sulphide-producing activity in MX-80 bentonite at 1750 and 2000 kg m− 3 wet density. SKB R-15–05.

[CR15] Berner UR (1992). Evolution of pore water chemistry during degradation of cement in a radioactive waste repository environment. Waste Manage.

[CR16] Bleyen N, Mariën A, Valcke E (2018). SCK•CEN/30259620: The geochemical perturbation of Boom Clay due to the NaNO3 plume released from Eurobitum bituminised radioactive waste: status 2013.

[CR17] Bowers KJ, Mesbah NM, Wiegel J (2009). Biodiversity of poly-extremophilic Bacteria: does combining the extremes of high salt, alkaline pH and elevated temperature approach a physico-chemical boundary for life?. Saline Systems.

[CR18] Brewitz W, Rothfuchs T (2007). Concepts and technologies for radioactive waste disposal in rock salt. Acta Montanistica Slovaca Ročník.

[CR19] Büppelmann K, Kim JI, Lierse C (1988). The redox-behaviour of plutonium in saline solutions under radiolysis effects. Radiochim Acta.

[CR20] Butterworth SJ, Stroes-Gascoyne S, Lloyd JR (2021). The microbiology of natural analogue sites. Microbiol Nuclear Waste Disposal.

[CR21] Caliò A, Dubois C, Fontanay S, Koza MM, Hoh F, Roumestand C, Oger P, Peters J (2022). Unravelling the adaptation mechanisms to high pressure in proteins. Int J Mol Sci.

[CR22] Charles CJ, Rout SP, Patel KA, Akbar S, Laws AP, Jackson BR, Boxall SA, Humphreys PN (2017). Floc formation reduces the pH stress experienced by microorganisms living in alkaline environments. Appl Environ Microbiol.

[CR23] Charles CJ, Rout SP, Laws AP, Jackson BR, Boxall SA, Humphreys PN (2017). The impact of biofilms upon surfaces relevant to an intermediate level radioactive waste geological disposal facility under simulated near-field conditions. Geosci Multidiscipl Digital Publ Inst.

[CR24] Charles CJ, Rout SP, Jackson BR, Boxall SA, Akbar S, Humphreys PN (2022). The evolution of alkaliphilic biofilm communities in response to extreme alkaline pH values. MicrobiologyOpen.

[CR25] Choi JY, Lee K, Lee PC (2019). Characterization of carotenoid biosynthesis in newly isolated Deinococcus sp. AJ005 and investigation of the effects of environmental conditions on cell growth and carotenoid biosynthesis. Mar Drugs.

[CR26] Coates JD, Michaelidou U, Bruce RA, O’Connor SM, Crespi JN, Achenbach LA (1999). ‘Ubiquity and diversity of dissimilatory (per)chlorate-reducing bacteria’, Applied and Environmental Microbiology. American Society for Microbiology.

[CR27] Costerton JW, Lewandowski Z, DeBeer D, Caldwell D, Korber D, James G (1994). Biofilms, the customized microniche. J Bacteriol.

[CR28] Daly MJ (2009). A new perspective on radiation resistance based on Deinococcus radiodurans. Nature Rev Microbiol Nature Publ Group.

[CR29] Daly MJ (2012). Death by protein damage in irradiated cells. DNA Repair Elsevier.

[CR30] Daly MJ, Ouyang L, Fuchs P, Minton KW (1994). In vivo damage and recA-dependent repair of plasmid and chromosomal DNA in the radiation-resistant bacterium Deinococcus radiodurans. J Bacteriol.

[CR31] Daly MJ, Gaidamakova EK, Matrosova VY, Vasilenko A, Zhai M, Venkateswaran A, Hess M, Omelchenko MV, Kostandarithes HM, Makarova KS, Wackett LP, Fredrickson JK, Ghosal D (2004). Accumulation of Mn(II) in Deinococcus radiodurans facilitates gamma-radiation resistance. Science.

[CR32] Daly MJ, Gaidamakova EK, Matrosova VY, Vasilenko A, Zhai M, Leapman RD, Lai B, Ravel B, Li SMW, Kemner KM, Fredrickson JK (2007). Protein oxidation implicated as the primary determinant of bacterial radioresistance. PLoS Biol.

[CR33] De Rezende JR, Kjeldsen KU, Hubert CRJ, Finster K, Loy A, Jørgensen BB (2013). Dispersal of thermophilic Desulfotomaculum endospores into Baltic Sea sediments over thousands of years. ISME J.

[CR34] Desouky O, Ding N, Zhou G (2015). ‘Targeted and non-targeted effects of ionizing radiation’. J Radiation Res Appl Sci.

[CR35] Edbeib MF, Wahab RA, Huyop F (2016). Halophiles: biology, adaptation, and their role in decontamination of hypersaline environments. World J Microbiol Biotechnol.

[CR36] Ewing RC, Weber WJ, Clinard FW (1995). Radiation effects in nuclear waste forms for high-level radioactive waste. Prog Nuclear Energy Pergamon.

[CR37] Fagliarone C, Mosca C, Ubaldi I, Verseux C, Baqué M, Wilmotte A, Billi D (2017). Avoidance of protein oxidation correlates with the desiccation and radiation resistance of hot and cold desert strains of the cyanobacterium Chroococcidiopsis. Extremophiles.

[CR38] Foster L, Boothman C, Ruiz-Lopez S, Boshoff G, Jenkinson P, Sigee D, Pittman JK, Morris K, Lloyd JR (2020). Microbial bloom formation in a high pH spent nuclear fuel pond. Sci Total Environ.

[CR39] Fredrickson JK, Zachara JM, Balkwill DL, Kennedy D, Li SMW, Kostandarithes HM, Daly MJ, Romine MF, Brockman FJ (2004). Geomicrobiology of high-level nuclear waste-contaminated vadose sediments at the Hanford Site, Washington State. Appl Environ Microbiol.

[CR40] Gaona X, Montoya V, Colàs E, Grivé M, Duro L (2008). Review of the complexation of tetravalent actinides by ISA and gluconate under alkaline to hyperalkaline conditions. J Contam Hydrol.

[CR41] Gilmour KA, Davie CT, Gray N (2021). An indigenous iron-reducing microbial community from MX80 bentonite—a study in the framework of nuclear waste disposal. Appl Clay Sci.

[CR42] Gilmour KA, Davie CT, Gray N (2022). ‘Survival and activity of an indigenous iron-reducing microbial community from MX80 bentonite in high temperature/low water environments with relevance to a proposed method of nuclear waste disposal’. Sci Total Environ.

[CR43] Glaus MA, Van Loon LR, Achatz S, Chodura A, Fischer K (1999). Degradation of cellulosic materials under the alkaline conditions of a cementitious repository for low and intermediate level radioactive waste Part I: identification of degradation products. Anal Chim Acta.

[CR44] Grigoryan, A., Wolfaardt, G. M., Stroes-Gascoyne, S., Keech, P., Jalique, D. R., McKelvie, J. and Korber, D. R. (2020) ‘Baseline distribution and diversity of MIC-relevant microorganisms in compacted bentonites after incubation for 1 year’, in *NACE - *International Corrosion Conference Series, pp. 1–5.

[CR45] Gruber C, Legat A, Pfaffenhuemer M, Radax C, Weidler G, Busse H-J, Stan-Lotter H (2004). Halobacterium noricense sp. nov., an archaeal isolate from a bore core of an alpine Permian salt deposit, classification of Halobacterium sp. NRC-1 as a strain of H. salinarum and emended description of H. salinarum. Extremophiles Springer-Verlag.

[CR46] Guo K, Cheng C, Chen L, Xie J, Li S, He S, Xiao F (2022). ‘Application of Deinococcus radiodurans in the treatment of environmental pollution by heavy metals and radionuclides’. J Radioanal Nuclear Chem.

[CR47] Hajj HE, Abdelouas A, Grambow B, Martin C, Dion M (2010). Microbial corrosion of P235GH steel under geological conditions. Phys Chem Earth.

[CR48] Hall DS, Behazin M, Jeffrey Binns W, Keech PG (2021). An evaluation of corrosion processes affecting copper-coated nuclear waste containers in a deep geological repository. Prog Mat Sci.

[CR49] Hammes F, Berney M, Egli T (2011). ‘Cultivation-independent assessment of bacterial viability’. Adv Biochem Eng/biotechnol.

[CR50] Haynes HM, Pearce CI, Boothman C, Lloyd JR (2018). ‘Response of bentonite microbial communities to stresses relevant to geodisposal of radioactive waste’. Chem Geol.

[CR51] Haynes HM, Bailey MT, Lloyd JR (2021). Bentonite barrier materials and the control of microbial processes: safety case implications for the geological disposal of radioactive waste. Chem Geol.

[CR52] Haynes, H. M., Nixon, S. and Lloyd, J. R. (2019) Verification of Microbial Sulfide-Producing Activity in Calcigel Bentonite at Wet Densities of 1750 and 1900 kg m-3.

[CR53] Hicks T, White M, Hooker P (2009). Role of bentonite in determination of thermal limits on geological disposal facility design.

[CR54] Hoehler TM, Jørgensen BB (2013). Microbial life under extreme energy limitation. Nature Rev Microbiol.

[CR55] Horikoshi K (2011). Extremophiles Handbook.

[CR56] Horn JM, Masterson BA, Rivera A, Miranda A, Davis MA, Martin S (2004). Bacterial growth dynamics, limiting factors, and community diversity in a proposed geological nuclear waste repository environment. Geomicrobiol J.

[CR57] Hou S, Larsen RW, Boudko D, Riley CW, Karatan E, Zimmer M, Ordal GW, Alam M (2000). Myoglobin-like aerotaxis transducers in Archaea and Bacteria. Nature.

[CR58] Hunsche U, Hampel A (1999). Rock salt—the mechanical properties of the host rock material for a radioactive waste repository. Eng Geol.

[CR59] Huttunen-Saarivirta E, Rajala P, Carpén L (2016). ‘Corrosion behaviour of copper under biotic and abiotic conditions in anoxic ground water: electrochemical study’. Electrochim Acta.

[CR60] Huttunen-Saarivirta E, Ghanbari E, Mao F, Rajala P, Carpén L, Macdonald DD (2018). Kinetic properties of the passive film on copper in the presence of sulfate-reducing bacteria. J Electrochem Soc.

[CR61] Itävaara M, Salavirta H, Marjamaa K, Ruskeeniemi T, Sariaslani S, Gadd GM (2016). Geomicrobiology and Metagenomics of Terrestrial Deep Subsurface Microbiomes. Advances in Applied Microbiology.

[CR62] Jebbar M, Franzetti B, Girard E, Oger P (2015). Microbial diversity and adaptation to high hydrostatic pressure in deep-sea hydrothermal vents prokaryotes. Extremophiles.

[CR63] Jin M, Xiao A, Zhu L, Zhang Z, Huang H, Jiang L (2019). ‘The diversity and commonalities of the radiation-resistance mechanisms of Deinococcus and its up-to-date applications’. AMB Express.

[CR64] Jørgensen BB, Boetius A (2007). Feast and famine-microbial life in the deep-sea bed. Nat Rev Microbiol.

[CR65] Jørgensen BB, Zawacki LX, Jannasch HW (1990). ‘Thermophilic bacterial sulfate reduction in deep-sea sediments at the Guaymas Basin hydrothermal vent site (Gulf of California)’. Deep Sea Res Part A Oceanographic Res Papers.

[CR66] Kashefi K, Holmes DE, Reysenbach AL, Lovley DR (2002). Use of Fe(III) as an electron acceptor to recover previously uncultured hyperthermophiles: isolation and characterization of Geothermobacterium ferrireducens gen nov., sp. nov. Appl Environ Microbiol.

[CR67] Kato C, Anitori RP (2012). Microbiology of Piezophiles in Deep-sea Environments. Extremophiles—Microbiology and Biotechnology.

[CR68] Kelm M, Bohnert E (2017). Radiation Chemical effects in the near field of a final Disposal Site–II: Simulation of the radiolytic processes in concentrated NaCl solutions. Nucl Technol.

[CR69] Kempes CP, van Bodegom PM, Wolpert D, Libby E, Amend J, Hoehler T (2017). Drivers of bacterial maintenance and minimal energy requirements. Front Microbiol.

[CR70] Kim J, Dong H, Seabaugh J, Newell SW, Eberl DD (2004). ‘Role of Microbes in the Smectite-to-Illite Reaction’. Science.

[CR71] Kosakowski, G., Berner, U., Wieland, E., Glaus, M. and Degueldre, C. (2014) *Geochemical Evolution of the L/ILW Near-field*, *Technical Report*.

[CR72] Kottemann M, Kish A, Iloanusi C, Bjork S, DiRuggiero J (2005). Physiological responses of the halophilic archaeon Halobacterium sp. strain NRC1 to desiccation and gamma irradiation. Extremophiles.

[CR73] Krulwich TA, Ito M, Gilmour R, Guffanti AA (1997). Mechanisms of cytoplasmic pH regulation in alkaliphilic strains of Bacillus. Extremophiles.

[CR74] Kuippers G, Bassil NM, Boothman C, Bryan N, Lloyd JR (2015). Microbial degradation of isosaccharinic acid under conditions representative for the far field of radioactive waste disposal facilities. Mineral Mag.

[CR75] Lennon JT, Jones SE (2011). Microbial seed banks: the ecological and evolutionary implications of dormancy. Nat Rev Microbiol.

[CR76] Leupin OX, Bernier-Latmani R, Bagnoud A, Moors H, Leys N, Wouters K, Stroes-Gascoyne S (2017). ‘Fifteen years of microbiological investigation in Opalinus Clay at the Mont Terri rock laboratory (Switzerland)’. Swiss J Geosci.

[CR77] Libert M, Bildstein O, Esnault L, Jullien M, Sellier R (2011). ‘Molecular hydrogen: an abundant energy source for bacterial activity in nuclear waste repositories’. Phys Chem Earth.

[CR78] Lopez-Fernandez M, Åström M, Bertilsson S, Dopson M (2018). Depth and dissolved organic carbon shape microbial communities in surface influenced but not ancient saline terrestrial aquifers. Front Microbiol.

[CR79] Lopez-Fernandez M, Broman E, Turner S, Wu X, Bertilsson S, Dopson M (2018). Investigation of viable taxa in the deep terrestrial biosphere suggests high rates of nutrient recycling. FEMS Microbiol Ecol.

[CR80] Marshall, M. H. M. and Simpson, M. J. (2014) *State of Science Review: Natural Organic Matter in Clays and Groundwater*. Toronto.

[CR81] Matschiavelli N, Kluge S, Podlech C, Standhaft D, Grathoff G, Ikeda-Ohno A, Warr LN, Chukharkina A, Arnold T, Cherkouk A (2019). The year-long development of microorganisms in uncompacted bavarian bentonite slurries at 30 and 60 °c. Environ Sci Technol.

[CR82] Mattimore V, Battista JR (1996). Radioresistance of Deinococcus radiodurans: Functions necessary to survive ionizing radiation are also necessary to survive prolonged desiccation. J Bacteriol.

[CR83] Merino N, Aronson HS, Bojanova DP, Feyhl-Buska J, Wong ML, Zhang S, Giovannelli D (2019). Living at the extremes: extremophiles and the limits of life in a planetary context. Front Microbiol.

[CR84] Mesbah NM, Hedrick DB, Peacock AD, Rohde M, Wiegel J (2007). Natranaerobius thermophilus gen. nov., sp. nov., a halophilic, alkalithermophilic bacterium from soda lakes of the Wadi An Natrun, Egypt, and proposal of Natranaerobiaceae fam. nov. and Natranaerobiales ord. nov. Int J Syst Evolut Microbiol.

[CR85] Miettinen H, Bomberg M, Bes R, Tiljander M, Vikman M (2022). Transformation of inherent microorganisms in Wyoming-type bentonite and their effects on structural iron. Appl Clay Sci.

[CR86] Milodowski AE, Shaw RP. and Stewart DI (2013) The Harpur Hill Site: its geology, evolutionary history and a catalogue of materials present, British Geological Survey Commissioned Report. Keyworth, Nottingham

[CR87] Mulligan CN, Yong RN, Fukue M (2009). Some effects of microbial activity on the evolution of clay-based buffer properties in underground repositories. Appl Clay Sci.

[CR88] NDA (2010) Geological Disposal: Near-field evolution status report, NDA Report. doi: NDA Report no. NDA/RWMD/036

[CR89] NDA (2016) NDA Report no DSSC/412/01 - Geological Disposal: Generic Disposal Facility Design

[CR90] NEA (2013) Underground Research Laboratories (URL) - NEA/RWM/R(2013)2

[CR91] NEA (2018) Microbial Influence on the Performance of Subsurface, Salt-Based Radioactive Waste Repositories, NEA No. 7387

[CR92] Newsome L, Morris K, Lloyd JR (2014). ‘The biogeochemistry and bioremediation of uranium and other priority radionuclides’. Chem Geol.

[CR93] Niedrée B, Vereecken H, Burauel P (2013). Radiation-induced impacts on the degradation of 2,4-D and the microbial population in soil microcosms. J Environ Radioact.

[CR94] Padan E, Bibi E, Ito M, Krulwich TA (2005). ‘Alkaline pH homeostasis in bacteria: new insights’,. Biochim Et Biophy Acta (BBA)—Biomembranes.

[CR95] Papini CM, Pandharipande PP, Royer CA, Makhatadze GI (2017). Putting the Piezolyte hypothesis under pressure. Biophys J.

[CR96] Park SH, Singh H, Appukuttan D, Jeong S, Choi YJ, Jung JH, Narumi I, Lim S (2017). PprM, a cold shock domain-containing protein from deinococcus radiodurans, confers oxidative stress tolerance to escherichia coli. Front Microbiol.

[CR97] Pedersen K (2002). Chapter 10 Microbial processes in the disposal of high level radioactive waste 500 m underground in Fennoscandian Shield rocks. Radioactivity Environ.

[CR98] Pedersen K (2010). Analysis of copper corrosion in compacted bentonite clay as a function of clay density and growth conditions for sulfate-reducing bacteria. J Appl Microbiol.

[CR99] Pedersen K, Motamedi M, Karnland O, Sandén T (2000). Cultivability of microorganisms introduced into a compacted bentonite clay buffer under high-level radioactive waste repository conditions. Eng Geol.

[CR100] Pentráková L, Su K, Pentrák M, Stucki JW (2013). A review of microbial redox interactions with structural Fe in clay minerals. Clay Miner.

[CR101] Perdrial JN, Warr LN, Perdrial N, Lett MC, Elsass F (2009). Interaction between smectite and bacteria: implications for bentonite as backfill material in the disposal of nuclear waste. Chem Geol.

[CR102] Podlech C, Matschiavelli N, Peltz M, Kluge S, Arnold T, Cherkouk A, Meleshyn A, Grathoff G, Warr LN (2021). Bentonite alteration in batch reactor experiments with and without organic supplements: implications for the disposal of radioactive waste. Minerals.

[CR103] Poolman B, Glaasker E (1998). Regulation of compatible solute accumulation in bacteria. Mol Microbiol.

[CR104] Potts M (1994). Desiccation tolerance of prokaryotes. Microbiol Rev Am Soc Microbiol.

[CR105] Rainey FA, Ray K, Ferreira M, Gatz BZ, Nobre MF, Bagaley D, Rash BA, Park MJ, Earl AM, Shank NC, Small AM, Henk MC, Battista JR, Kämpfer P, Da Costa MS (2005). Extensive diversity of ionizing-radiation-resistant bacteria recovered from Sonoran Desert soil and description of nine new species of the genus Deinococcus obtained from a single soil sample. Appl Environ Microbiol.

[CR106] Reiller P, Moulin V, Casanova F, Dautel C (2002). Retention behaviour of humic substances onto mineral surfaces and consequences upon thorium (IV) mobility: case of iron oxides. Appl Geochem Pergamon.

[CR107] Rizoulis A, Steele HM, Morris K, Lloyd JR (2012). The potential impact of anaerobic microbial metabolism during the geological disposal of intermediate-level waste. Mineral Mag.

[CR108] Robb FT, Clark DS (1999). Adaptation of proteins from hyperthermophiles to high pressure and high temperature. J Mol Microbiol Biotechnol.

[CR109] Rout SP, Charles CJ, Doulgeris C, McCarthy AJ, Rooks DJ, Loughnane JP, Laws AP, Humphreys PN (2015). Anoxic biodegradation of isosaccharinic acids at alkaline pH by natural microbial communities. PLoS ONE.

[CR110] Salin ML, Brown-Peterson NJ (1993). Dealing with active oxygen intermediates: A halophilic perspective. Experientia.

[CR111] Schu MK, Schlegel ML, Libert M, Bildstein O (2015). Impact of iron-reducing bacteria on the corrosion rate of carbon steel under simulated geological disposal conditions. Environ Sci Technol.

[CR112] Scoma A, Garrido-Amador P, Nielsen SD, Røy H, Kjeldsen KU (2019). The polyextremophilic bacterium clostridium paradoxum attains piezophilic traits by modulating its energy metabolism and cell membrane composition. Appl Environ Microbiol.

[CR113] Sellin P, Leupin OX (2014). ‘The use of clay as an engineered barrier in radioactive-waste management—a review’. Clays Clay Minerals.

[CR114] Shuryak I, Matrosova VY, Gaidamakova EK, Tkavc R, Grichenko O, Klimenkova P, Volpe RP, Daly MJ (2017). Microbial cells can cooperate to resist high-level chronic ionizing radiation. PLoS ONE.

[CR115] Siliakus MF, Van Der Oost J, Servé, · and Kengen, W. M.  (2017). Adaptations of archaeal and bacterial membranes to variations in temperature, pH and pressure. Extremophiles.

[CR116] SKB (1999) SR 97, Processes in the Repository Evolution, SKB Report TR-99–07.

[CR117] Small JS, Nykyri M, Vikman M, Itävaara M, Heikinheimo L (2017). The biogeochemistry of gas generation from low-level nuclear waste: modelling after 18 years study under in situ conditions. Appl Geochem.

[CR118] Sterner R, Liebl W (2001). Thermophilic adaptation of proteins. Crit Rev Biochem Mol Biol.

[CR119] Stockdale A, Davison W, Zhang H (2009). Micro-scale biogeochemical heterogeneity in sediments: a review of available technology and observed evidence. Earth-Sci Rev.

[CR120] Sutherland IW (2001). Biofilm exopolysaccharides: a strong and sticky framework. Microbiology.

[CR121] Takai K, Nakamura K, Toki T, Tsunogai U, Miyazaki M, Miyazaki J, Hirayama H, Nakagawa S, Nunoura T, Horikoshi K (2008). Cell proliferation at 122°C and isotopically heavy CH4 production by a hyperthermophilic methanogen under high-pressure cultivation. Proc Natl Acad Sci United States of Am.

[CR122] Torres E, Turrero MJ, Moreno D, Sánchez L, Garralón A (2017). FEBEX In-Situ test: preliminary results of the geochemical characterization of the metal/bentonite interface. Procedia Earth Planet Sci.

[CR123] Towler G, Bond AE, Watson S, Norris S, Suckling P, Benbow S (2012). Understanding the behaviour of gas in a geological disposal facility: modelling coupled processes and key features at different scales. Mineral Magazine Mineral Soc.

[CR124] Tsang C-F, Bernier F, Davies C (2005). Geohydromechanical processes in the Excavation Damaged Zone in crystalline rock, rock salt, and indurated and plastic clays-in the context of radioactive waste disposal. Int J Rock Mech Min Sci.

[CR125] Vachon MA, Engel K, Beaver RC, Slater GF, Binns WJ, Neufeld JD (2021). Fifteen shades of clay: distinct microbial community profiles obtained from bentonite samples by cultivation and direct nucleic acid extraction. Scient Rep Nature Publ Group UK.

[CR126] Wang H, Zhang Y, Bartlett DH, Xiao X (2021). Transcriptomic analysis reveals common adaptation mechanisms under different stresses for moderately piezophilic bacteria. Micr Ecol.

[CR127] Webb KM, Yu J, Robinson CK, Noboru T, Lee YC, DiRuggiero J (2013). Effects of intracellular Mn on the radiation resistance of the halophilic archaeon Halobacterium salinarum. Extremophiles.

[CR128] Williamson AJ, Morris K, Shaw S, Byrne JM, Boothman C, Lloyd JR (2013). Microbial reduction of Fe(III) under alkaline conditions relevant to geological disposal. Appl Environ Microbiol.

[CR129] Yin W, Wang Y, Liu L, He J (2019). Biofilms : the microbial “ protective clothing ” in extreme environments. Int J Mol Sci.

[CR130] Youngblut MD, Wang O, Barnum TP, Coates JD (2016). (Per)chlorate in biology on earth and beyond. Annu Rev Microbiol.

[CR131] Younger PL, Boyce AJ, Waring AJ (2015). Chloride waters of Great Britain revisited: from subsea formation waters to onshore geothermal fluids. Proc Geologists.

[CR132] Zavarzin GA, Zhilina TN, Kevbrin VV (1999). The alkaliphilic microbial community and its functional diversity. Microbiology.

[CR133] Zhang G, Huihua G, Yi L (2013). Stability of halophilic proteins: From dipeptide attributes to discrimination classifier. Int J Biol Macromol.

